# Genomic Surveillance Enables Suitability Assessment of *Salmonella* Gene Targets Used for Culture-Independent Diagnostic Testing

**DOI:** 10.1128/JCM.00038-20

**Published:** 2020-08-24

**Authors:** Rebecca J. Rockett, Alicia Arnott, Qinning Wang, Peter Howard, Vitali Sintchenko

**Affiliations:** a Sydney Medical School, The University of Sydney, Sydney, New South Wales, Australia; b Centre for Infectious Diseases and Microbiology–Public Health, Westmead Hospital, Westmead, New South Wales, Australia; c Institute of Clinical Pathology and Medical Research, NSW Health Pathology, Westmead, New South Wales, Australia; National Institute of Allergy and Infectious Diseases

**Keywords:** culture-independent diagnostic testing, pathogen genomics, *Salmonella*, public health

## Abstract

*Salmonella* is a highly diverse genus consisting of over 2,600 serovars responsible for high-burden food- and waterborne gastroenteritis worldwide. Sensitivity and specificity of PCR-based culture-independent diagnostic testing (CIDT) systems for *Salmonella*, which depend on a highly conserved gene target, can be affected by single nucleotide polymorphisms (SNPs), indels, and genomic rearrangements within primer and probe sequences. This report demonstrates the value of prospectively collected genomic data for verifying CIDT targets. We utilized the genomes of 3,165 *Salmonella* isolates prospectively collected and sequenced in Australia. The sequences of *Salmonella* CIDT PCR gene targets (*ttrA*, *spaO*, and *invA*) were systematically interrogated to measure nucleotide dissimilarity. Analysis of 52 different serovars and 79 multilocus sequencing types (MLST) demonstrated dissimilarity within and between PCR gene targets ranging between 0 and 81.3 SNP/kbp (0 and 141 SNPs). The lowest average dissimilarity was observed in the *ttrA* target gene used by the Roche LightMix at 2.0 SNP/kbp (range, 0 to 46.7); however, entropy across the gene demonstrates that it may not be the most stable CIDT target. While debate continues over the benefits and pitfalls of replacing bacterial culture with molecular assays, the growing volumes of genomic surveillance data enable periodic regional reassessment and validation of CIDT targets against both prevalent and emerging serovars. If PCR systems are to become the primary screening and diagnostic tool for laboratory diagnosis of salmonellosis, ongoing monitoring of the genomic diversity in PCR target regions is warranted, as is the potential inclusion of two *Salmonella* PCR targets in frontline diagnostic systems.

## Abstract (precis)

*Salmonella* is a highly diverse genus consisting of over 2,600 serovars responsible for high-burden food- and waterborne gastroenteritis worldwide. Sensitivity and specificity of PCR-based culture-independent diagnostic testing (CIDT) systems for *Salmonella*, which depend on a highly conserved gene target, can be affected by single nucleotide polymorphisms (SNPs), indels, and genomic rearrangements within primer and probe sequences. This report demonstrates the value of prospectively collected genomic data for verifying CIDT targets.

## INTRODUCTION

*Salmonella* is the leading cause of foodborne gastroenteritis worldwide, resulting in significant morbidity, mortality, and economic cost (1). *Salmonella* is a highly diverse genus of zoonotic organisms, serologically classified into over 2,600 serovars. The genus *Salmonella* is divided into two major species, Salmonella enterica and Salmonella bongori. S. enterica is considered the most pathogenic, as the genome contains both *Salmonella* pathogenicity islands 1 and 2 (SPI1 and SPI2). S. enterica is further differentiated into six subspecies: S. enterica subsp. *enterica* (subspecies I), S. enterica subsp. *salamae* (II), S. enterica subsp. *arizonae* (IIIa), S. enterica subsp. *diarizonae* (IIIb), S. enterica subsp. *houtenae* (IV), and S. enterica subsp. *indica* (VI). Nontyphoidal *Salmonella* (NTS) infections are primarily caused by serovars within subspecies I, with S. enterica subspecies *enterica* serovar Typhimurium being a leading cause of foodborne outbreaks in many countries, including Australia. However, over 200 other serovars have also been reported as causal agents of NTS, including some that are unique to Australia (2, 3). The prevalence of *Salmonella* serovars differs significantly between continents. For example, S. enterica subspecies *enterica* serovar Enteritidis is the most commonly detected serovar in Europe and North America, whereas *S*. Typhimurium predominates in Australia and New Zealand (4–7).

The public health, food safety, and trade implications of foodborne *Salmonella* are such that a highly sensitive and specific surveillance system is required to ensure rapid detection and characterization of foodborne outbreaks. The introduction and often complete replacement of traditional culture for *Salmonella* with culture-independent diagnostic testing (CIDT) as the front-end method of detecting *Salmonella* in stool samples has presented several challenges to public health laboratory surveillance (5, 8–10). The reliance of all current CIDT platforms on a single gene target for the detection of a diverse pathogen such as *Salmonella* has raised important questions regarding ability of these CIDTs to detect uncommon serovars or emerging variants. As CIDTs become more widely used for *Salmonella* diagnosis, they become, in some cases, the only signal of a potential outbreak and the need for reflex culture. Therefore, any loss in CIDT sensitivity can have serious consequences for the diagnosis of individual cases as well as recognition and management of outbreaks.

Although PCR-based techniques are considered extremely sensitive, the accumulation of mutations within PCR targets has been documented for other pathogens. Mutations or indels can occur within CIDT primer and probe target regions, and depending on the location of these mutations, CIDT sensitivity can be diminished, in some cases resulting in false-negative results. For example, the sole reliance on CIDT to diagnose the highly conserved bacterium Chlamydia trachomatis is an example of the shortcomings of CIDT-only testing algorithms (11). In 2006, it was reported that a C. trachomatis variant was circulating and that this variant harbored a deletion in the PCR primer target region contained within the cryptic plasmid (11). This highly conserved multicopy plasmid had been thought to be the ideal diagnostic target, as it offered increased sensitivity for detecting C. trachomatis. However, emergence of a variant harboring a deletion within the cryptic plasmid caused a complete loss of sensitivity in the majority of commercial CIDTs, resulting in the ongoing transmission of C. trachomatis and an increased number of severe sequalae after unrecognized and prolonged C. trachomatis infections (12). These false-negative results for patients with C. trachomatis variant infections were noted only after a significant decrease in the incidence of C. trachomatis triggered an investigation by public health authorities. The response to this CIDT system failure was to design an additional C. trachomatis PCR target. The vulnerability of CIDT to generation of false-negative results due to nucleotide dissimilarity has led to the recommendation that a two-target system is needed for all frontline infectious disease CIDT assays (13). Despite this, the current *Salmonella* CIDTs are often reliant on a single PCR target region.

Whole-genome sequencing (WGS) provides the ultimate resolution to correctly identify outbreak clusters and detect food or environmental pathogen reservoirs (14, 15). This ability triggered the rapid uptake of WGS as the preferred approach for public health surveillance of salmonellosis, resulting in accumulation of genome sequence data. These genomic data provide an opportunity to examine the variability of molecular targets employed by different CIDT platforms in the context of locally circulating *Salmonella* serovars. In this study, we examined the variability in CIDT targets and the robustness of current CIDT systems utilizing a comprehensive *Salmonella* genome collection spanning more than two summer seasons in New South Wales (NSW), Australia.

## MATERIALS AND METHODS

### Isolate collections.

*Salmonella* isolates included in this study represent all isolates referred to the NSW Enteric Reference Laboratory, Institute of Clinical Pathology and Medical Research (ICPMR), NSW Pathology, that underwent whole-genome sequencing as part of routine public health outbreak investigations in NSW, Australia between October 2015 and December 2018 (*n* = 3,256). In addition, WGS was performed on 43 isolates collected from historical outbreaks of salmonellosis where the causal *Salmonella* serovar had previously been described as native to Australia (3).

### Nucleic acid extraction and library preparation.

A single colony was used for DNA extraction; extraction was performed using the Geneaid Presto genomic DNA bacterial kit (Geneaid, Taiwan) per the manufacturer’s instructions for Gram-negative bacteria. DNA extracts were treated with 1 U of RNase. DNA libraries were prepared with the Nextera XT library preparation kit, using 1 ng/μl of DNA in accordance with manufacturer’s instructions. Multiplexed libraries were sequenced using paired-end 150-bp chemistry on the NextSeq 500 system (Illumina, Australia).

### Bioinformatic analysis of sequenced genomes.

Demultiplexed sequencing reads with >1 × 10^7^ reads per isolate were trimmed (16), based on a minimum quality read score of 20, and then *de novo* assembled using SPAdes (version 3.13.0) (17). The quality of *de novo* assemblies was assessed with Quast (version 5.0.2) (18); only assemblies with <200 contigs and *N*_50_ values of >50,000 bp were included in further analysis. MLST and serovar were inferred from final contigs using the Salmonella enterica pubMLST scheme (https://github.com/tseemann/mlst) and SISTR (19). Contigs were annotated with Prokka (version 1.13.3) (20). Core genome analysis was conducted using Roary (the pangenome pipeline; version 3.12.0) (21). Maximum-likelihood phylogeny of the pangenome was generated using the general time-reversible (GTR+R4) model with IQ-TREE (version 1.6.3) (22). Phylogeny and metadata were visualized with Microreact (23) and Inkscape.

CIDT gene targets were extracted from contigs using in-house Perl scripts and BLAST+ (24). CIDT target genes from the closed reference genome Salmonella enterica subsp. *enterica* serovar Typhimurium strain LT2 (GenBank accession no. AE006468.2) were used as a reference. All CIDT nucleotide sequences extracted from isolate contigs were aligned to the reference using MAFFT (25). Single nucleotide polymorphisms (SNPs) were called by their comparison to the reference genome using SNP-sites (26). SNP differences and the length of PCR target genes were used to calculate the dissimilarity index (number of SNPs per kilobase pair) for each gene (gene lengths: *invA*, 2,057 bp; *spaO*, 919 bp; and *ttrA*, 3,062 bp). Entropy at each position within PCR target genes was measured from the PCR gene target alignments using the formula *H*(*l*) = ∑*f*(*b*,*l*)log_(base 2)_ *f*(*b*,*l*).

### Primer and probe design and assessment.

The specific nucleotide sequences of the primer and probes used by the commercial CIDTs investigated in this study are proprietary; however, the genes they target have been reported. To investigate diversity with the primer and probe sequences, we performed an *in silico* prediction of primer and probe targets for each gene. High-homology regions of the PCR target genes (*ttrA*, *spaO*, and *invA*) were extracted from reference genomes of 10 common medically relevant serovars associated with salmonellosis (*S*. Typhimurium, *S*. Enteritidis, *S*. Newport, *S*. Saintpaul, *S*. Virchow, *S*. Infantis, *S*. Heidelberg, *S*. Montevideo, *S*. Javiana, *S*. Muenchen, and *S*. Braenderup). The reference Salmonella enterica genomes for these serovars were downloaded, and target gene sequences were extracted as outlined in the bioinformatic analyses described above (the NCBI accession numbers for reference genomes are AE006468.2, CP045063, NC_011080.1, NC_011083.1, NC_011294.1, NC_020307.1, NZ_CP007530.1, NZ_CP017727.1, NZ_CP022490.1, NZ_CP025094.1, and NZ_LN649235.1). Regions of homology between all sequences >300 bp in length were employed to find the best primer and probe targets using the PrimerQuest tool (Integrated DNA Technologies). The predicted oligonucleotides were then extracted from the genomes used in this study to investigate polymorphisms within the primer and probe sequences. The impact of any polymorphisms detected was based on both the number of SNPs in each oligonucleotide and the position of the SNPs within the oligonucleotide. SNPs within the last 5 bp of the 3′ end of the oligonucleotide were considered to have a moderate effect on PCR sensitivity, as were more than two SNPs within the same oligonucleotide. Significant sensitivity losses are predicted to occur when more than three SNPs are detected within the same oligonucleotide or more than one SNP within the last 5 bp at the 3′ end of each oligonucleotide. Limited sensitivity losses were predicted for single SNPs outside the last 5 bp at the 3′ end of the primer or probe (27).

### Statistical analysis.

The diversity of NTS serovars in Australia between 2009 and 2017 was investigated using the National Notifiable Diseases Surveillance System Public *Salmonella* data set (http://www9.health.gov.au/cda/source/pub_salmo.cfm). The total number of NTS serovars reported and case notifications of salmonellosis each year were used to calculate a Simpson’s index of diversity.

### Data availability.

The assembled genomes for all 3,165 isolates and individual gene target sequences *ttrA*, *spaO*, and *invA* were deposited in the NCBI (BioProject no. PRJNA596817). Associated metadata, including genome assembly statistics, multilocus sequence typing (MLST) results, and serovar prediction, can be found in Data Set S1 in the supplemental material.

## RESULTS

### Phylogenomic comparison of *Salmonella* genomes.

**FIG 1:**
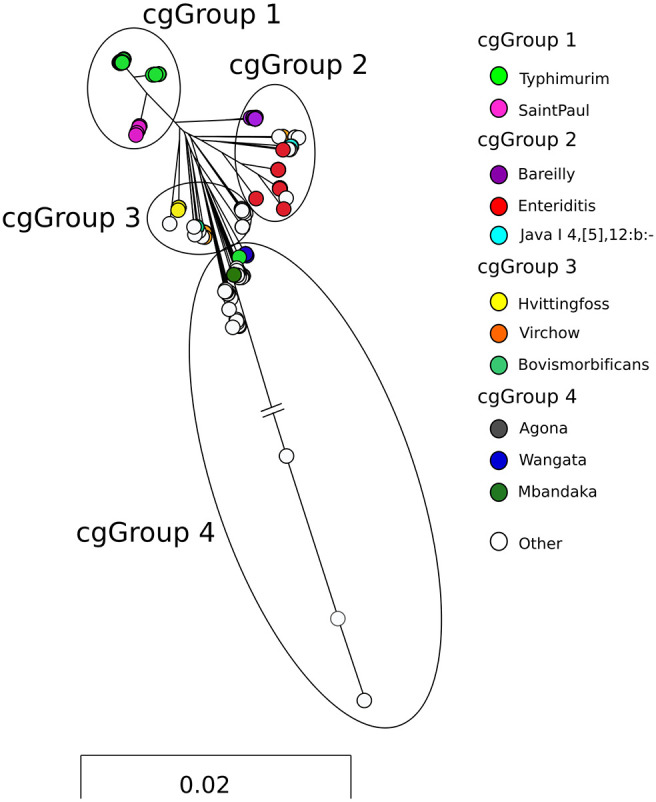
Maximum-likelihood phylogeny constructed from 3,270 core *Salmonella* genes. Colored nodes represent serovars which contain more than 20 isolates within the phylogeny; white nodes represent serovars with fewer than 20 isolates. Long branches of cgGroup 4 have been truncated to aid visualization.

A total of 3,165 *Salmonella* isolates were included in this study. Isolate genomes that failed to meet sequence quality metrics or were sequenced multiple times were removed (*n* = 134). The core genome shared by study isolates consisted of 3,270 genes with a total length of 2,963,964 bp. Maximum-likelihood core genome phylogeny was constructed using 98,254 polymorphic sites (median SNP distance, 13,588; range, 0 to 98,254) within core genes and demonstrated branching largely corresponding to serovar differentiation (Fig. 1). The core genome included the PCR gene targets investigated in this study: *ttrA*, *spaO*, and *invA*. Assembly statistics for *Salmonella* genomes included in the study are outlined in Table S1. The genomes investigated represented 52 different serovars and 79 MLST types. The most common serovar was *S*. Typhimurium (*n* = 2,175; 69%), including the monophasic *S*. Typhimurium variant I with the antigenic structure 4,[5],12:i:− (*n* = 212; 7%), followed by *S*. Enteritidis (*n* = 406; 13%) and *S*. Saintpaul (*n* = 97; 3%). A detailed list of the serovars and MLST types of all isolates is presented in Table S2.

To examine CIDT target nucleotide diversity, we divided the genomes into four groups (core genome groups [cgGroups] 1 to 4) according to the phylogenetic analysis (Fig. 1), with the aim of determining if CIDT target diversity correlated with core genome diversity. cgGroup 1 contained 2,271 genomes and was dominated by the most common serovar in Australia, *Salmonella* Typhimurium (including monophasic *S*. Typhimurium; *n* = 2,174), in addition to *S*. Saintpaul (*n* = 97). cgGroup 2 consisted primarily of 515 genomes belonging to *S*. Enteritidis (*n* = 406), *S*. Bareilly (*n* = 46), and *S*. Java (antigenic structure, I 4,[5],12:b:−) (*n* = 53). cgGroup 3 included 183 genomes and was composed primarily of *S*. Hvittingfoss (*n* = 106), *S*. Virchow (*n* = 36), and *S*. Bovismorbificans (*n* = 17). cgGroup 4 contained 196 genomes representing largely *S*. Wangata (*n* = 90), *S*. Mbandaka (*n* = 40), and *S*. Agona (*n* = 23). Long branches are attributed to *Salmonella* isolates within S. enterica subsp. *houtenae*, S. enterica subsp. *diarizonae*, and S. enterica subsp. *salamae* (Fig. 1); branch lengths in the figure have been truncated to ease illustration of all core genome groups. cgGroup 4 also includes serovars that are unique to Australia (*S*. Adelaide, *S*. Chester, *S*. Orion, and *S*. Tennessee). A detailed list of genomes included in this study with corresponding serovars, MLST types, assembly metrics, and cgGroup information can be found in Table S2.

### Nucleotide dissimilarity of PCR target genes.

**FIG 2:**
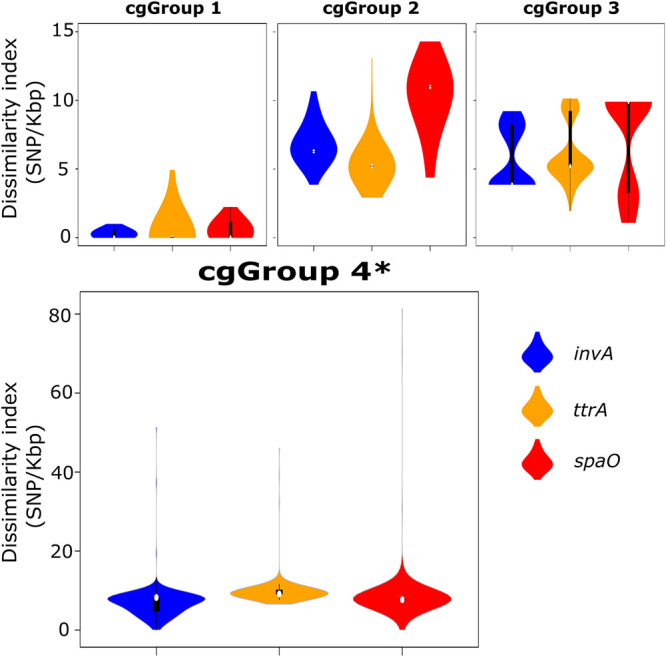
Nucleotide dissimilarity of CIDT PCR targets within each of the four phylogenetic groups. *, note the increased dissimilarity scale for cgGroup4 genomes.

Significant nucleotide diversity was identified within all PCR target regions (Fig. 2). Dissimilarity of CIDT targets within the same serovar was low. For example, the median dissimilarity indexes (range) between *S. Typhimurium* isolates within *invA*, *ttrA*, and *spaO* nucleotide sequences in group 1 of our set were 0.27 (0 to 1), 0 (0 to 10.8), and 1.1 (0 to 1.1), respectively. In contrast, significant dissimilarity was noted between other cgGroups containing a higher diversity of serovars. For the *invA* region, the median nucleotide dissimilarity indexes (range) were 6.8 (3.9 to 9.7), 3.9 (3.9 to 9.2), and 8.2 (0 to 51.3) for cgGroups 2, 3, and 4, respectively; for *ttrA*, dissimilarity indexes were 5.2 (3.9 to 10.5), 5.2 (2 to 13.1), and 9.1 (6.5 to 46.1); and for *spaO*, they were 10.9 (1.1 to 12.1), 9.9 (1.1 to 13.2), and 8.6 (0 to 81.3), respectively. The lowest median dissimilarity between all *Salmonella* genomes was within the *ttrA* gene. Interestingly, the highest dissimilarity indexes were observed within genes from *Salmonella* genomes in cgGroup 4, which included a representative from each of the S. enterica subspecies II to IV (S. enterica subsp. *salamae*, S. enterica subsp. *diarizonae*, and S. enterica subsp. *houtenae*).

### Entropy of CIDT target genes.

**FIG 3:**
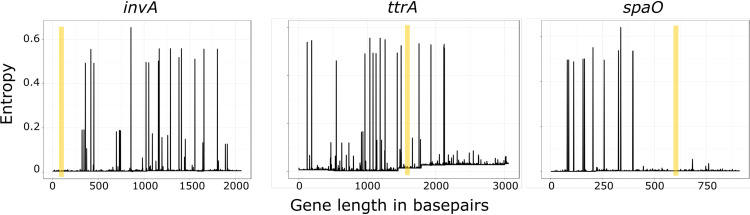
Entropy across the nucleotide sequence of the three CIDT targets, *invA*, *ttrA*, and *spaO*. Yellow boxes highlight the optimal primer and probe target regions within each gene.

Entropy of CIDT target regions indicated that nucleotide dissimilarity was dispersed across the length of the sequence (Fig. 3). Clear regions of conservation were seen in the 3′ end of *invA* and 5′ end of *spaO* gene sequences. However, polymorphisms were present throughout the *ttrA* nucleotide sequence. In addition, 16 genomes had truncations of the 5′ and 3′ ends of the *ttrA* nucleotide sequence (median length, 1,337 bp [range, 60 to 3,035 bp]). However, these genomes had lower-quality assembly metrics than the entire genome collection. The medium number of contigs in the eight assemblies that contained a truncated *ttrA* gene sequence was 99.5 (34 to 135), in contrast to the median number for all genomes analyzed, 78 (24 to 169). *N*_50_ values for genomes containing a truncated *ttrA* nucleotide sequence, i.e., 139,974 (66,538 to 261,187), were slightly lower than those for all genomes analyzed, i.e., 172,206 (52,145 to 518,076). Therefore, these truncations may have resulted from *de novo* assembly errors.

### Nucleotide diversity in primer and probe targets.

Optimal primer and probe sequences were predicted *in silico* using regions of PCR target genes homologous among 10 common Salmonella enterica serovars. Predicted gene sequences and oligonucleotide parameters are specified in Table 1. Entropy across the reverse transcription-PCR (RT-PCR) amplicon for each gene target is depicted in Fig. 3. When the entropy of the CIDT target was compared across all genomes in this study, the RT-PCR target regions remained in areas of low nucleotide variability. The specific oligonucleotide sequences were extracted from each genome, with only 2% (60/3,165) of genomes containing SNPs within proposed oligonucleotide sequences. Four, 12, and 44 genomes contained oligonucleotide mutations in the *invA*, *ttrA*, and *spaO* RT-PCR oligonucleotides, respectively. Most single SNPs in either the forward or reverse primers and probe oligonucleotides were expected to have a small impact on assay sensitivity (48/60). However, the remaining 12 genomes had multiple SNPs within a single oligonucleotide or contained a single mutation in multiple oligonucleotides. Multiple mutations in single oligonucleotides or mutations across all oligonucleotides of an RT-PCR are likely to lead to significant sensitivity losses and in some cases false-negative results. The genomes harboring multiple mutations (12/60) were all contained within cgGroup 4, which contained the most divergent core genomes (Table 2).

**TABLE 1:** Oligonucleotide sequences and PCR parameters

Oligonucleotide	Sequence (5′–3′)	*T_m_*	% GC	Amplicon length (bp)
invA_forward	CACTGACTTGCTATCTGCTATCTC	62	45.8	126
invA_reverse	AGGAGGACAAGATCTTTATGTGC	62	43.5	
invA_probe	AAATCGACGGACATCGACAGACGT	67	50.0	
ttrA_forward	GCTTACCGAACTGTTGACCTC	63	52.4	100
ttrA_reverse	TTTACGGTGTTCCCGGTCTA	63	50.0	
ttrA_probe	CGCTCGAAGGCTATCCTTATCCGC	68	58.0	
spaO_forward	TCCCGTGCGGAAGTTTATTG	63	50.0	146
spaO_reverse	GAAACTCTGCCTGGCTTGAA	63	50.0	
spaO_probe	CGCTCGAAGGCTATCCTTATCCGC	67	48.0	

**TABLE 2:** Polymorphisms in RT-PCR assay oligonucleotide sequences, with predicted loss in assay sensitivity

Gene	Oligonucleotide affected	Position (base) in oligonucleotide from 5′ end	Predicted loss in sensitivity	Serovar(s) (total no. of genomes encoding that serovar)	Total no. of genomes	Core genome group
*invA*	Forward primer	16	Moderate	Untyped (1), ST237	1	4
	Forward primer	1 (deletion)	Limited	II1,4,[5],12,[27] (1)^*a*^	1	4
	Probe	1	Limited	Clanvillian (1)^*a*^	1	4
	Reverse primer	12	Limited	IIIb,50:k:z35 (1)^*a*^; II1,4,[5],12,[27] (1)^*a*^	2	4

*ttrA*	Forward primer	5	Limited	Enteritidis (469)	1	2
	Forward primer	4	Limited	Untyped (1), ST237	1	4
	Forward primer	6	Limited	Senftenberg (2), ST210	1	4
	Probe	2 SNPs, 2 and 11	Moderate	Tennessee (3), ST319	3	4
	Probe	11	Limited	Havana (4), ST595, ST578	3	4
	Probe	20	Moderate	Typhimurium (2175), ST19	1	1
	Probe	1	Limited	Untyped (1), ST237	1	4
	Reverse primer	14	Limited	Untyped (1), ST237	1	4

*spaO*	Forward primer	4 and 12	Moderate	IIIb,50:k:z35 (1)^*a*^	1	4
	Reverse primer	12	Limited	Mbandaka (40), ST413	40	4
	Reverse primer	4, 12, and 15	Significant	II1,4,[5],12,[27] (1)^*a*^	1	4
	Reverse primer	3, 5, and 15	Significant	Untyped (1), ST237	1	4
	Reverse primer	5 and 15	Moderate	IIIb,50:k:z35 (1)^*a*^	1	4

a This is a novel allele combination; therefore, the ST could not be determined.

### Temporal trends in Australian nontyphoidal *Salmonella* serovar diversity.

**FIG 4:**
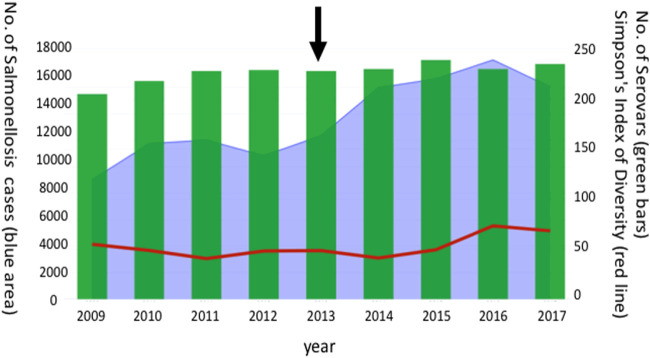
Serotype diversity of notified cases of salmonellosis in Australia between 2009 and 2017. CIDT platforms became available in 2013 in Australia (arrow). Simpson’s index of diversity was multiplied by a factor of 50 to aid visual representation on the graph.

Based on the Australian National Notifiable Diseases Surveillance System public *Salmonella* data set, there were more than 200 serovars identified in association with 14,000 salmonellosis cases during each year during the period from 2009 to 2017 in Australia. A gradual increase in serotype diversity and reported incidence of nontyphoidal salmonellosis was detected between 2009 and 2017 (Fig. 4). CIDTs for *Salmonella* diagnosis were introduced widely across Australia in late 2013, and notable increases in the Simpson’s diversity index were observed after 2015. Subspecies II to IV account for around 1% of salmonellosis cases reported each year in Australia.

## DISCUSSION

WGS is being increasingly utilized to investigate outbreaks of *Salmonella* and to accurately identify food and environmental sources of infection. This high-resolution approach has greatly enhanced the speed and accuracy of public health interventions ensuring food safety. Genome sequencing data collected as part of prospective public health surveillance also enable the ongoing sensitivity of foodborne outbreak surveillance to be determined and monitored, as described in the present study. Public health authorities have raised concerns that the increasing reliance on CIDTs as the only tool to detect pathogens responsible for foodborne disease will significantly reduce the sensitivity of public health surveillance systems, as serotyping and WGS require *Salmonella* to be isolated by solid medium culture (8, 9, 28). The replacement of conventional culture with CIDT reduces the number of isolates available for WGS and therefore the ability of public health laboratories to identify and control outbreaks of salmonellosis. In Australia, it has been reported that 12 to 18% of *Salmonella*-positive stool samples are identified based solely on CIDT testing (8, 29). In North America, *Salmonella*-positive stool samples are often reflexively cultured after a positive *Salmonella* CIDT at public health laboratories in an attempt to boost the number of *Salmonella* isolates available for WGS; Australia has not yet widely adopted reflex culture after a positive *Salmonella* CIDT; however, it may be required to maintain the current WGS-based surveillance systems (5, 10).

CIDT platforms offer highly automated “swab-to-result” systems with high throughput, rapid turnaround time, and reduced testing costs. However, most commercial assays offer a single PCR target for *Salmonella* detection, with the specific primer and probe target sequences being difficult to uncover, as they are commonly proprietary. In this study, we systematically interrogated three genes used as PCR targets by different CIDTs, i.e., *ttrA* (Roche LightMix), *spaO* (BD Max), and *invA* (a common in-house *Salmonella* PCR target). While our genomic analysis based on WGS data allowed the assessment of dissimilarity of CIDT targets, the proprietary nature of PCR primers and probes made it difficult to accurately estimate the extent of the potential sensitivity losses. We were, however, reassured that significant changes in the spectrum and diversity of *Salmonella* serovars do not appear to have occurred despite the recent introduction of CIDT systems for detection of bacterial enteropathogens in stool samples from patients with gastroenteritis in Australia. The Australian market has been dominated by commercial systems, namely, the BD Max enteric bacterial panel PCR assay and the Roche LightMix fecal PCR screening kit. The recent implementation of other panels, including the BioFire FilmArray GI panels, for which the gene targets are not disclosed, as well as noncommercial PCR CIDT, has only recently occurred within the Australian diagnostic testing landscape, and hence, their impact on *Salmonella* surveillance is yet to be determined. If proprietary CIDT primer and probe nucleotide sequences are not made freely available, increased diligence from companies and pathology providers may be required, with expanded evaluation of CIDT targets and ongoing monitoring of diversity within CIDT targets. Without this reassurance from test providers, monitoring trends in the diversity of *Salmonella* serovars is important to monitor the impact of CIDT, particularly as an array of different enteric CIDT panels are added to the diagnostic capacity in Australia.

Dissimilarity of PCR targets occurred within each of the 52 *Salmonella* serovars investigated; the highest levels of dissimilarity were noted among the three genomes falling outside S. enterica subsp. *enterica* (subspecies I). Although *Salmonella* subspecies I isolates predominate as the causal agents of foodborne outbreaks in Australia and elsewhere, it should be noted that current CIDTs may have diminished sensitivity to detect the less common S. enterica subspecies with potential for emergence as human pathogens, especially isolates from the predominantly zoonotic subspecies IV and VI (24). Among the individual genes, entropy across PCR targets suggests that the *spaO* gene may contain a more conserved PCR target region, while other targets may be affected by dissimilarity across the entire gene. In particular, truncations of the *ttrA* gene region were present in a small number of genomes. The *ttrA* gene encodes tetrathionate reductase subunit A and is part of the *ttrRSBCA* operon, which is required for tetrathionate respiration and located in close proximity to *Salmonella* pathogenicity island 2. The integration of virulence factors or other evolutionary pressures may increase the variability in this particular CIDT target, but these truncations will need to be validated in other data sets to exclude bioinformatic assembly errors.

Truncations aside, the effect of polymorphisms in oligonucleotides on assay sensitivity is difficult to assess and can be affected by a number of parameters. The largest sensitivity losses are seen with mutations at the 3′ end of primer sequences (30). However, the composition of the mismatched base also plays a role, as purine-pyrimidine mismatches are generally less detrimental than purine-purine/pyrimidine-pyrimidine polymorphisms (27, 31, 32). More general considerations are also important, including the master mix composition and the number of multiplexed primer and probe combinations. Generally, the more primer pairs are included in a multiplex reaction, the greater the loss of sensitivity for the mismatched primer set, particularly in cases involving polymicrobial intestinal infections. These observations are especially relevant and important, as prediction models indicate that even a small loss of PCR sensitivity could be detrimental to outbreak detection.

In conclusion, the growing volumes of genomic surveillance data allow ongoing reassessment and validation of CIDT targets representing prevalent and emerging serotypes of public health significance. Nucleotide dissimilarity of CIDT targets in different serovars of *Salmonella* subspecies IV and VI may affect the public health surveillance of nontyphoidal salmonellosis in areas where they are endemic. If CIDT systems are to become the primary screening and diagnostic tool for laboratory diagnosis of salmonellosis, ongoing monitoring of the local genomic diversity in PCR target regions is warranted. A two-gene target detection system is also recommended and would limit the potential for false-negative CIDT results for *Salmonella*.

## Supplementary Material

Supplemental file 1

Supplemental file 2

## References

[1] Kirk MD, Pires SM, Black RE, Caipo M, Crump JA, Devleesschauwer B, Döpfer D, Fazil A, Fischer-Walker CL, Hald T, Hall AJ, Keddy KH, Lake RJ, Lanata CF, Torgerson PR, Havelaar AH, Angulo FJ 2015 World Health Organization estimates of the global and regional disease burden of 22 foodborne bacterial, protozoal, and viral diseases, 2010: a data synthesis. PLoS Med 12:e1001921. doi:10.1371/journal.pmed.1001921. 26633831 PMC4668831

[2] Ashbolt R, Kirk MD 2006 Salmonella Mississippi infections in Tasmania: the role of native Australian animals and untreated drinking water. Epidemiol Infect 134:1257–1265. doi:10.1017/S0950268806006224. 16672107 PMC2870509

[3] Iveson JB, Bradshaw SD, Smith DW 2017 The movement of humans and the spread of Salmonella into existing and pristine ecosystems. Microbiol Aust 38:201–203. doi:10.1071/MA17070.

[4] Centers for Disease Control and Prevention. 2016 National enteric disease surveillance: Salmonella annual report, 2016. Centers for Disease Control and Prevention, Atlanta, GA.

[5] Berenger B, Chui L, Reimer AR, Allen V, Alexander D, Domingo M-C, Haldane D, Hoang L, Levett P, MacKeen A, Marcino D, Sheitoyan-Pesant C, Zahariadis G, Canadian Public Health Laboratory Network. 2017 Canadian Public Health Laboratory Network position statement: non-culture based diagnostics for gastroenteritis and implications for public health investigations. Can Commun Dis Rep 43:279–281. doi:10.14745/ccdr.v43i12a06. 29770061 PMC5764713

[6] European Food Safety Authority. 2018 The European Union summary report on trends and sources of zoonoses, zoonotic agents and food‐borne outbreaks in 2017. EFSA J 16:5500. 10.2903/j.efsa.2018.5500 PMC7009540 32625785

[7] The Institute of Environmental Science and Research Ltd. 2019 Notifiable diseases in New Zealand: annual report 2017. Institute of Environmental Science and Research Ltd., Porirua, New Zealand.

[8] May FJ, Stafford RJ, Carroll H, Robson JM, Vohra R, Nimmo GR, Bates J, Kirk MD, Fearnley EJ, Polkinghorne BG 2017 The effects of culture independent diagnostic testing on the diagnosis and reporting of enteric bacterial pathogens in Queensland, 2010 to 2014. Commun Dis Intell Q Rep 41:E223–E230. 29720071 10.33321/cdi.2017.41.32

[9] Shea S, Kubota KA, Maguire H, Gladbach S, Woron A, Atkinson-Dunn R, Couturier MR, Miller MB 2017 Clinical microbiology laboratories’ adoption of culture-independent diagnostic tests is a threat to foodborne-disease surveillance in the United States. J Clin Microbiol 55:10–19. doi:10.1128/JCM.01624-16. 27795338 PMC5228220

[10] Tack DM, Marder EP, Griffin PM, Cieslak PR, Dunn J, Hurd S, Scallan E, Lathrop S, Muse A, Ryan P, Smith K, Tobin-D'Angelo M, Vugia DJ, Holt KG, Wolpert BJ, Tauxe R, Geissler AL 2019 Preliminary incidence and trends of infections with pathogens transmitted commonly through food—Foodborne Diseases Active Surveillance Network, 10 U.S. Sites, 2015–2018. MMWR Morb Mortal Wkly Rep 68:369–373. doi:10.15585/mmwr.mm6816a2. 31022166 PMC6483286

[11] Ripa T, Nilsson P 2006 A variant of Chlamydia trachomatis with deletion in cryptic plasmid: implications for use of PCR diagnostic tests. Euro Surveill 11:E061109.2. doi:10.2807/esw.11.45.03076-en. 17213548

[12] Dahlberg J, Hadad R, Elfving K, Larsson I, Isaksson J, Magnuson A, Fredlund H, Unemo M, Herrmann B 2018 Ten years transmission of the new variant of Chlamydia trachomatis in Sweden: prevalence of infections and associated complications. Sex Transm Infect 94:100–104. doi:10.1136/sextrans-2016-052992. 28724744 PMC5870454

[13] Whiley DM, Lambert SB, Bialasiewicz S, Goire N, Nissen MD, Sloots TP 2008 False-negative results in nucleic acid amplification tests—do we need to routinely use two genetic targets in all assays to overcome problems caused by sequence variation? Crit Rev Microbiol 34:71–76. doi:10.1080/10408410801960913. 18568861

[14] Besser JM 2018 Salmonella epidemiology: a whirlwind of change. Food Microbiol 71:55–59. doi:10.1016/j.fm.2017.08.018. 29366469

[15] Sintchenko V, Holmes EC 2015 The role of pathogen genomics in assessing disease transmission. BMJ 350:h1314. doi:10.1136/bmj.h1314. 25964672

[16] Bolger AM, Lohse M, Usadel B 2014 Trimmomatic: a flexible trimmer for Illumina sequence data. Bioinformatics 30:2114–2120. doi:10.1093/bioinformatics/btu170. 24695404 PMC4103590

[17] Bankevich A, Nurk S, Antipov D, Gurevich AA, Dvorkin M, Kulikov AS, Lesin VM, Nikolenko SI, Pham S, Prjibelski AD, Pyshkin AV, Sirotkin AV, Vyahhi N, Tesler G, Alekseyev MA, Pevzner PA 2012 SPAdes: a new genome assembly algorithm and its applications to single-cell sequencing. J Comput Biol 19:455–477. doi:10.1089/cmb.2012.0021. 22506599 PMC3342519

[18] Gurevich A, Saveliev V, Vyahhi N, Tesler G 2013 QUAST: quality assessment tool for genome assemblies. Bioinformatics 29:1072–1075. doi:10.1093/bioinformatics/btt086. 23422339 PMC3624806

[19] Yoshida CE, Kruczkiewicz P, Laing CR, Lingohr EJ, Gannon VPJ, Nash JHE, Taboada EN 2016 The Salmonella In Silico Typing Resource (SISTR): an open web-accessible tool for rapidly typing and subtyping draft Salmonella genome assemblies. PLoS One 11:e0147101. doi:10.1371/journal.pone.0147101. 26800248 PMC4723315

[20] Seemann T 2014 Prokka: rapid prokaryotic genome annotation. Bioinformatics 30:2068–2069. doi:10.1093/bioinformatics/btu153. 24642063

[21] Page AJ, Cummins CA, Hunt M, Wong VK, Reuter S, Holden MTG, Fookes M, Falush D, Keane JA, Parkhill J 2015 Roary: rapid large-scale prokaryote pan genome analysis. Bioinformatics 31:3691–3693. doi:10.1093/bioinformatics/btv421. 26198102 PMC4817141

[22] Nguyen LT, Schmidt HA, Von Haeseler A, Minh BQ 2015 IQ-TREE: a fast and effective stochastic algorithm for estimating maximum-likelihood phylogenies. Mol Biol Evol 32:268–274. doi:10.1093/molbev/msu300. 25371430 PMC4271533

[23] Argimón S, Abudahab K, Goater RJE, Fedosejev A, Bhai J, Glasner C, Feil EJ, Holden MTG, Yeats CA, Grundmann H, Spratt BG, Aanensen DM 2016 Microreact: visualizing and sharing data for genomic epidemiology and phylogeography. Microb Genom 2:e000093. 28348833 10.1099/mgen.0.000093 PMC5320705

[24] Camacho C, Coulouris G, Avagyan V, Ma N, Papadopoulos J, Bealer K, Madden TL 2009 BLAST+: architecture and applications. BMC Bioinformatics 10:421–429. doi:10.1186/1471-2105-10-421. 20003500 PMC2803857

[25] Katoh K, Standley DM 2013 MAFFT multiple sequence alignment software version 7: improvements in performance and usability. Mol Biol Evol 30:772–780. doi:10.1093/molbev/mst010. 23329690 PMC3603318

[26] Keane JA, Page AJ, Delaney AJ, Taylor B, Seemann T, Harris SR, Soares J 2016 SNP-sites: rapid efficient extraction of SNPs from multi-FASTA alignments. Microb Genom 2:e000056. doi:10.1099/mgen.0.000056. 28348851 PMC5320690

[27] Lefever S, Pattyn F, Hellemans J, Vandesompele J 2013 Single-nucleotide polymorphisms and other mismatches reduce performance of quantitative PCR assays. Clin Chem 59:1470–1480. doi:10.1373/clinchem.2013.203653. 24014836

[28] Cronquist AB, Mody RK, Atkinson R, Besser J, D’Angelo MT, Hurd S, Robinson T, Nicholson C, Mahon BE 2012 Impacts of culture-independent diagnostic practices on public health surveillance for bacterial enteric pathogens. Clin Infect Dis 54:S432–S439. doi:10.1093/cid/cis267. 22572666

[29] Communicable Diseases Branch. 2019 OzFoodNet NSW Annual Report: 2018. Health Protection NSW, Sydney, Australia.

[30] Whiley DM, Sloots TP 2005 Sequence variation in primer targets affects the accuracy of viral quantitative PCR. J Clin Virol 34:104–107. doi:10.1016/j.jcv.2005.02.010. 16157260

[31] Klungthong C, Chinnawirotpisan P, Hussem K, Phonpakobsin T, Manasatienkij W, Ajariyakhajorn C, Rungrojcharoenkit K, Gibbons RV, Jarman RG 2010 The impact of primer and probe-template mismatches on the sensitivity of pandemic influenza A/H1N1/2009 virus detection by real-time RT-PCR. J Clin Virol 48:91–95. doi:10.1016/j.jcv.2010.03.012. 20413345

[32] McLauchlin J, Aird H, Andrews N, Chattaway M, de Pinna E, Elviss N, Jørgensen F, Larkin L, Willis C 2019 Public health risks associated with Salmonella contamination of imported edible betel leaves: analysis of results from England, 2011–2017. Int J Food Microbiol 298:1–10. doi:10.1016/j.ijfoodmicro.2019.03.004. 30889473

