# Supplementary material for: Genomic Surveillance Enables Suitability Assessment of Salmonella Gene Targets Used for Culture-Independent Diagnostic Testing
Source: J Clin Microbiol. 2020 Aug 24;58(9):e00038-20. doi: 10.1128/JCM.00038-20 (PMC7448617; doi:10.1128/JCM.00038-20)
Supplement: Supplemental file 1 [file JCM.00038-20-s0002.pdf]

Supplementary Material

Supplemental Table S1. *de novo* assembly metrics of *Salmonella* genomes

***de novo* assembly metrics**	**Median (range)**
**Number of contigs (>1000bp)**	78 (24-169)
**Genome size**	4,879,273 (4,185,374 - 5,283,669)
**GC content (%)**	52.2 (51.5 - 54.5)
**N50**	172,206 (52,145 - 518,076)
**N75**	75607 (27,194 - 253,326)

Supplemental Table S2. Predicted *Salmonella* Serovar, MLST and core genome group of isolates

Salmonella Serovar	*cgGroup*	*ST*	*Isolate numbers*
Agona	Group 4	13	23
Bareilly	Group 2	203	41
	Group 2	909	5
Bovismorbificans	Group 3	377	16
	Group 3	1499	1
Enteritidis	Group 2	10	1
	Group 2	11	301
	Group 2	74	1
	Group 2	180	33
	Group 2	1925	58
	Group 2	1972	2
	Group 2	3233	4
	Group 2	3304	5
	Group 2	Undetermined	1
Hvittingfoss	Group 3	434	94
	Group 3	446	3
	Group 3	2062	10
Mbandaka	Group 4	413	40
Saintpaul	Group 1	27	3
	Group 1	50	151
	Group 1	Undetermined	1
Typhimurium 4,[5],12:i:-	Group 1	34	218
Typhimurium	Group 1	19	1922
	Group 1	36	31
	Group 1	99	2
	Group 1	313	2
	Group 1	2066	3
	Group 1	2089	5
	Group 1	2297	1

	Group 1	Undetermined	39
I 4, [5],12:B:-	Group 2	2358	1
	Group 2	4959	52
Wangata	Group 4	523	90
Virchow	Group 3	16	35
	Group 3	197	1
	Group 4	359	1

* Serovars with less than 20 genomes included; Aberdeen (n=1, ST426, cgGroup 3), Abortusovis (n=4, ST768, cgGroup 2), Adelaide (n=4, ST440, cgGroup 4), Agoueve (n=1, ST286, cgGroup 4), Apeyeme (n=1, ST1546, cgGroup 4), Bahrenfeld (n=1, ST859, cgGroup 4), Birkenhead (n=1, ST424, cgGroup 3), Broughton (n=1, Undetermined, cgGroup 4), Cerro (n=1, ST3548, cgGroup 4), Chester (n=5, ST343, n=1 Undetermined, cgGroup 4), Clanvillian (n=1, Undetermined cgGroup 2), Galiema (n=1, ST601, cgGroup 3), Gallinarum (n=1, ST92 cgGroup 2), Orion (n=6, ST580 (n=2), ST639 (n=3), cgGroup 4), Give (n=1, ST516, cgGroup 4), Havana (n=4, (ST578, n=3, ST595, n=1, cgGroup 4), II 1,4,[5],12,[27]:b:[e,n,x] (n=1, ST2116, cgGroup 4), IIIb 50:k:z35 (n=1, Undetermined, cgGroup 4), Infantis (n=7, ST32, cgGroup 3), Litchfield (n=1, ST4491, cgGroup 3), Manchester (n=1, ST491, cgGroup 3), Mgulani (n=1, ST2033, cgGroup 3), Minnesota (n=1, ST548 cgGroup 4), Mishmarhaemek (n=1, ST2666, cgGroup 3), Montevideo (n=1, Undetermined, cgGroup 3), Muenchen (n=5, (ST82, n=3, ST112, n=1, ST3211, n=1, cgGroup 3)), Newport (n=1, ST31 cgGroup 3), Oesterbro (n=1, ST466, cgGroup 3), Oranienburg (n=1, ST23, cgGroup 4), Orientalis (n=1, ST558, cgGroup 4), Paratyphi A (n=1, ST129, cgGroup 4), Potsdam (n=1, ST2462 (n=1 cgGroup 2), Poona (n=1, ST1069, cgGroup 3), Senftenberg (n=2, (ST185, ST210, cgGroup 4), Singapore (n=2, (ST462, n=1, cgGroup 3), (ST501, n=1, cgGroup 4)), Stanley (n=2, (ST29, n=1), (ST2299, n=1) , cgGroup 2, Tennessee (n=3, ST319, cgGroup 4), and Typhi (n=2, ST1, cgGroup 4)
